# How to Make Feedback More Effective? Qualitative Findings from Pilot Testing of an Audit and Feedback Report for Endoscopists

**DOI:** 10.1155/2016/4983790

**Published:** 2016-09-18

**Authors:** Fiona Webster, Jigisha Patel, Kathleen Rice, Nancy Baxter, Lawrence Paszat, Linda Rabeneck, Jill Tinmouth

**Affiliations:** ^1^Department of Health Policy, Management and Evaluation, University of Toronto, Toronto, ON, Canada; ^2^Dalla Lana School of Public Health, University of Toronto, Toronto, ON, Canada; ^3^Sunnybrook Health Sciences Centre, Toronto, ON, Canada; ^4^Department of Family and Community Medicine, University of Toronto, Toronto, ON, Canada; ^5^Institute for Clinical Evaluative Sciences, Toronto, ON, Canada; ^6^Department of Surgery and Li Ka Shing Knowledge Institute, St. Michael's Hospital, Toronto, ON, Canada; ^7^Department of Medicine, University of Toronto, Toronto, ON, Canada; ^8^Cancer Care Ontario, Toronto, ON, Canada

## Abstract

*Background*. Audit and feedback (A/F) reports are one of the few knowledge translation activities that can effect change in physician behavior. In this study, we pilot-tested an endoscopist A/F report to elicit opinions about the proposed report's usability, acceptability and usefulness, and implications for knowledge translation.* Methods*. Semi-structured qualitative interviews were conducted with eleven endoscopists in Ontario, Canada. We tested an A/F report template comprising 9 validated, accepted colonoscopy quality indicators populated with simulated data. Interview transcripts were coded using techniques such as constant comparison and themes were identified inductively over several team meetings.* Results*. Four interrelated themes were identified: (1) overall perceptions of the A/F report; (2) accountability and consequences for poor performance; (3) motivation to change/improve skills; and (4) training for performance enhancement and available resources. The A/F report was well received; however, participants cited some possible threats to the report's effectiveness including the perceived threat of loss of privileges or licensing and the potential for the data to be dismissed.* Conclusions*. Participants agreed that A/F has the potential to improve colonoscopy performance. However, in order to be effective in changing physician behavior, A/F must be thoughtfully implemented with attention to the potential concerns of its recipients.

## 1. Introduction

Audit and feedback (A/F) is widely used as a strategy to improve professional practice and is one of the more effective knowledge translation strategies that can effect a change in physician behavior [1]. A/F involves providing a physician with a summary of their clinical performance over a specified period of time with the goal of promoting improvement in practice [2]. It is intended to provide an objective measure of a physicians' performance compared to their peers and jurisdictional targets [3]. The ultimate goal is to prompt physicians to take action and modify their performance if their clinical practice is identified as suboptimal [2].

Improving physician performance can be challenging and may be influenced by the method of feedback and the characteristics of the targeted behavior [4, 5]. A recent systematic review of 140 A/F trials [2] reported that multifaceted and complementary interventions such as the addition of reminders [6] and incentives [7] were more beneficial than A/F alone. Furthermore, the literature suggests that feedback may be more effective when baseline performance is low, the source is a supervisor or colleague, it is provided more than once, it is delivered in both verbal and written formats, and it includes both explicit goals and an action plan [2]. Changing complex physician behavior, such as a procedural skill, may present greater challenges than changing simpler behaviors such as prescribing or test-ordering [2]. Perhaps because of the complex nature of colonoscopy practice, there are relatively few trials of A/F reports for colonoscopy.

High quality colonoscopy is integral to the prevention and diagnosis of colorectal cancer (CRC). Globally, CRC is the 3rd leading cause of cancer-related death among men and women [8]. Unfortunately, colonoscopy quality is highly variable [9, 10] and poor quality colonoscopy adversely affects patients as it is associated with increased morbidity and mortality. Variation in the quality of colonoscopy is likely multifactorial; however, variation in endoscopist performance is clearly an important contributor.

In prior research, we determined the most accurate definitions of key colonoscopy quality indicators from Ontario health administrative data. Cancer Care Ontario (CCO) recently updated its Guideline for Colonoscopy Quality Assurance in Ontario [11] which identifies evidence-based endoscopist quality indicators for colonoscopy. In the current study, we developed an endoscopist A/F report template to measure colonoscopy performance based on these quality indicators. The objective of this study was to pilot-test the endoscopist A/F report to elicit opinions about the proposed report's usability, acceptability, and potential usefulness for knowledge translation.

## 2. Methods

### 2.1. Participants and Setting

We undertook a qualitative research study [12]. Participants were purposively sampled using a maximum variation strategy to ensure that they were at different stages in their careers, employed in a range of health care settings across Ontario, and diverse in terms of age and sex. A key recruitment resource was an expert panel on colonoscopy struck by CCO in order to advise on the development of a colonoscopy quality management program. These endoscopists were approached to participate because of their considerable expertise and leadership in this field.

Recruitment, development of interview materials, data collection, and analysis were collaboratively carried out by an interdisciplinary research team which consisted of a gastroenterologist with an academic affiliation (J. T.), a medical sociologist (F. W.), a medical anthropologist (K. R.), and a research coordinator (J. P.). The study was approved by the Research Ethics Board at Sunnybrook Health Sciences Centre in Toronto, Ontario.

### 2.2. Audit and Feedback Report

An endoscopist A/F report template was developed that incorporated nine colonoscopy quality indicators: annual colonoscopy volume, polypectomy rate (by sex), cecal intubation rate (by sex), polypectomy-associated bleeding rate, number of perforations, CRC detection rate, poor bowel preparation rate, postcolonoscopy CRC rate, and early repeat colonoscopy rate. CCO's Guideline for Colonoscopy Quality Assurance [11] was the primary resource for these indicators. An information sheet with definitions and methodology used to derive the indicators was included as supplementary material and was provided to participants during the interview. For the purposes of the current study, one sample report was populated with simulated data; however, participants were informed that these reports could be generated centrally using health administrative data (i.e., populated with real data) in the future for all endoscopists in the province of Ontario. All participants provided feedback on the same sample report.

### 2.3. Data Collection

Data were collected through semi-structured, one-on-one interviews that took place either in person or by telephone (with a web conferencing component) between April and July 2013. All participants signed an informed consent form prior to the interviews and received an honorarium for their time.

An interview guide was developed and pilot-tested prior to commencing the study. All interviews were approximately one hour in length and were led by a moderator experienced in qualitative research (K. R.). Feedback was obtained on the content and usability of the A/F report, preliminary impressions of the acceptability of the report, and how recipients might translate knowledge from the report into improving their endoscopy practice. Sample size was determined by saturation, when the team collaboratively decided that no new information or themes were being generated, at which point participant recruitment and interviewing stopped [13].

### 2.4. Data Analysis

Interviews were audio-recorded, transcribed verbatim, and analyzed using standard qualitative strategies including those from constructivist grounded theory approaches. Constructivist grounded theory refers to an approach to qualitative research that recognizes the researcher's role in the co-construction of meaning during interviews and in the multiplicity of meanings that events may have for participants [14]. Therefore, reflexivity is an important part of the research process, from design to analysis. Transcripts were imported into a qualitative software program (NVivo 10, QSR International Pty Ltd.). The constant comparison method was utilized; that is, each new transcript was compared with previous interview data. Using this approach, the verbatim text was coded line by line by two qualitative analysts (K. R. and J. P.). The analysts independently coded the first two transcripts, met to compare their interpretations, and developed a coding framework. The remaining transcripts were divided and each analyst coded half. The analysts categorically merged the codes; codes were then organized into categories and themes were constructed. Several team meetings were held over the duration of the data analysis phase, to discuss the implications and possible interpretations of the data. The regular meetings provided an opportunity for reflexive sharing and ensured rigor. Research validity was enhanced as the analytic approach incorporated the different disciplinary perspectives of the research team.

## 3. Results

In total, 11 endoscopists in Ontario were interviewed. Three (27%) were women, 82% had been in practice for over 15 years, 64% were gastroenterologists, 36% were general surgeons, and 55% had community-based practices. Seven (64%) participants were members of the Ontario colonoscopy expert panel struck by CCO (Table 1).

Our original questions probed the concepts of usability, acceptability, and usefulness but our interpretation of the data suggests that other factors may be important in terms of how the A/F report might be used. Four main themes emerged from the interviews: (1) overall perceptions of the A/F report; (2) accountability and consequences for poor performance; (3) motivation to change or improve skills; and (4) training for performance enhancement and available resources. In addition to the quotes below, a table of supplementary quotes for the 4 themes is in Supplementary Material available online at http://dx.doi.org/10.1155/2016/4983790.

*Overall Perceptions of the A/F Report*. The A/F approach was well received and participants welcomed feedback on their performance. The initiative was considered important because it provides individual endoscopist-level performance feedback as well as standards against which performance could be measured. Participants discussed the importance of having accurate information which could be used to benchmark performance.

*…I think [reports] are extremely valuable, particularly…to give an individual their performance versus provincial average. You know, versus expectations is one thing, but I think versus your peers is very valuable, and I think for all of us we want to do a good job and so that feedback's very valuable in terms of assessing our own performance. It doesn't necessarily always tell you what you need to change or work on but I think that feedback's very valuable.* (General surgeon, Interviewee #4)

Despite the consensus around the importance of receiving feedback, some participants raised concerns that low-performing endoscopists might challenge the data's accuracy.

*…So one of the reactions if somebody tells you your measurements are off is you usually say, “Well, look, the measurement tool's not very good, is it, really?”* (Gastroenterologist, Interviewee #11)

Some participants also expressed their preferences for data presentation that is meaningful and relevant. In particular, peer comparison to those with a similar practice and patient mix was emphasized.

*I'd like to compare myself to other people with a similar practice, not the people who see 90% screening colonoscopies in outpatient clinic…So some bit of demographics on the physicians I'm being compared to, because I would expect that I might get more complications or I might get less cecal intubation rates or something….* (Gastroenterologist, Interviewee #9)

*Accountability and Consequences for Poor Performance*. There was considerable disagreement as to how the A/F reports should be used and to what extent the information should be made public. Opinions on the extent of disclosure ranged from keeping the data highly confidential to making it fully public, seemingly reflecting a tension between the threat to the individual endoscopist's professional standing and transparency. Participants endorsing the former opinion were concerned about the impact the report may have on endoscopists' hospital privileges and licensing and suggested that the report should at least initially be confidential with the goal of enhancing performance only. Participants began to differentiate between individuals having information to assess their own performance “compared to peers” versus data that could be used by administrators or regulators, with the attendant possible threats, such as loss of privileges.

*…The big fear people probably have is if this goes to your chief of department who sees that your rates aren't as good [as] somebody else's, is it going to [affect] your privileging…is the licensing body going to get particularly concerned about it? I think all of us want to know how we're doing compared to our peers and getting that feedback confidentially.* (General surgeon, Interviewee #4)

Not all participants were apprehensive about sharing information with the public despite the potential negative impact described above. It was suggested that individual endoscopists were accountable for their own clinical performance. These participants focused on the possible consequences of poor quality colonoscopy for patients who they felt should be “more informed.”

*I think…if I want a colonoscopy done I'm going to go to someone I think can do a good job, and so I think patients are becoming more informed….* (Gastroenterologist, Interviewee #6)

Some participants felt that the A/F data should be reported to other stakeholders such as hospital or endoscopy unit administrators. They believed that since endoscopists' privileges are granted and maintained at individual facilities, these facilities are accountable for the quality of care they provide and facility administrators should be made aware of individual endoscopists' reports. Administrators could then use these data to inform clinicians that they “gotta get fixed.”

*…And if they're working in a unit, from my point of view the unit needs to have a report card and the unit needs to report that back to the College whether it's going to be at a hospital unit or in a private endoscopy unit, so this is my report card for all my people and I as a medical director will look at my report card, say, “You know what? You gotta get fixed. We'll help you fix, this is what we're going to do, but you need to go get fixed or you can't do this.”* (Gastroenterologist, Interviewee #8)

Interestingly, some participants linked preferred remedies for poor performance with the endoscopists' stage in their career. Some suggested that younger endoscopists would benefit from further training to improve their performance, while end-of-career endoscopists should consider retiring from their practice altogether.

*And especially if it's a younger person…or a mid-tier person who has…a significant career ahead of them and they're otherwise good…they just need a little bit more coaching/mentoring/training, then we should invest the time to…put them on that track and help them…So I guess the bottom 25% we have to decide, you know, is this the kind of person that just needs some extra work, or is it time for them to get out of the colonoscopy business….* (Gastroenterologist, Interviewee #1)

*Motivation to Change or Improve Skills*. Participants were asked if they believed most endoscopists would be motivated to change/improve their skills if they received a poor report. Responses varied and this variation seemed to be linked to participants' perceptions of how self-motivated clinicians were. Participants were mainly optimistic that the motivation to change would be high because they perceived that, as a group, endoscopists tend to be high performing and self-motivated. They hypothesized that they would be positively motivated to change/improve their skills. Receiving a low score would therefore induce a desire to improve.

*…Now, remember, most people that are in surgery and medicine…they're used to be considered high-performing individuals, right…So, you know, most of these folks kind of went through school and they were always at the top tier, they don't want to be at the bottom tier….* (Gastroenterologist, Interviewee #1)

Given the perception that there is a natural tendency for endoscopists to be high performers, many believed that the process of improving skills should be voluntary rather than mandatory and that at least initially performance change should rely on endoscopists' personal motivation to improve.

*They may not be happy – no one's happy to get negative feedback – but they should be aware of it and hopefully would do something about it…I think this process should be voluntary…Maybe after five years of being in the lower 25% there could be some discussion, but for the first couple of years I think it should be up to the physician to pull their own socks up.* (Gastroenterologist, Interviewee #5)

It was suggested that a certain subgroup of endoscopists, consisting of older and end-of-career endoscopists, might be the most challenging group with respect to improving their skills. Because of the stage in their career, several participants assumed that this subgroup would be less motivated to improve.

*You know, the problem is it's going to end up being, for the most part, a certain group of endoscopists in a certain demographic – they're going to be surgically trained, and they're going to be older…and often retired from hospital practice…That is going to be the biggest group that's going to have the worst numbers, and to date there's not been a way to deal with those people.* (Gastroenterologist, Interviewee #8)

*Training for Performance Enhancement and Available Resources*. Several participants identified a gap in terms of the resources currently available for additional training/skill enhancement for those identified as low performing. Most participants agreed that they were not aware of existing educational opportunities and would not know where to begin if they wanted to improve their colonoscopy performance.

*Yeah, that's a good question, because I think the average [endoscopist] ha[s] no idea where to go.* (Gastroenterologist, Interviewee #2)

Some felt that it was therefore imperative to provide resources to assist with improvement along with the A/F report. They suggested that information about interventions for skills enhancement could be provided with the report in order to facilitate the process of translating knowledge from the report into improving practice.

*Now, in the first go-round putting out the data's probably okay, but then we have to be thinking very…strategically what are we going to do when we keep giving these scorecards, right? Like, what are we going to do to help people. Because the next response would be, you know, people are saying, “You know what? Thanks a lot, but where do I go?” and you say, “Well, tough bananas, we don't really have anything for you,” that's [not] a good thing.* (Gastroenterologist, Interviewee #1)

Participants underscored the importance of having networks and colleagues with whom one could share poor scores and suggested that access to networks and/or training might vary by jurisdiction. One participant noted that being “recently out of training” enabled them to still access those who trained them in seeking help, suggesting that trainees or recent graduates would find it easier to seek and to get help.

*It's a little easier for me, maybe, because I'm recently out of training so I still have a network of mentors that I talk to all the time. I would start with my own colleagues at my hospital but I would also go back to the people who train me and say, “Wait a minute, I'm screwing up!” I think it might be a little more challenging for someone who'd been out of practice for fifteen/twenty years to know where to go, though.* (General surgeon, Interviewee #3)

## 4. Discussion

The A/F report was well received and the participants in our study confirmed that A/F has the potential to be an effective quality improvement strategy [4]. However, participants also cited some possible threats to the report's effectiveness including the perceived threat of loss of privileges or licensing, the potential for the data to be perceived as inaccurate, and the general lack of awareness of available resources for skill enhancement.

Participants suggested that underperforming physicians would likely challenge the data, either by rejecting the data or by suggesting that their practice differs importantly from their peers, rendering the reports' comparisons unfair and meaningless. This finding highlights one of the challenges of providing feedback to physicians—the concept of cognitive dissonance [15], which refers to the situation where an individual struggles with holding two conflicting beliefs simultaneously. Using the A/F reports as an example, for some physicians, acceptance of the data in the report would mean acknowledging that they are underperforming. However, this acceptance would likely conflict with the physician's self-perception that they are capable and competent practitioners. In order to resolve the conflict, physicians will likely find it easier to dismiss the data than question their own competency. A growing body of evidence suggests physicians are generally poor at self-assessment and tend to overestimate rather than underestimate their own performance [16–18]. Physicians' poor self-assessment skills may further push them to resolve cognitive dissonance by dismissing the data. It has been suggested that if coaching accompanies the feedback of “objective” data such as that found in our report, it may help the recipient interpret the data and set goals while maintaining their sense of self as competent practitioners [15].

An important consequence of dismissing the data is that the recipient's attention will be shifted away from the “task,” in this case colonoscopy performance. According to Kluger and DeNisi's feedback intervention theory [19], a key characteristic of effective feedback is that it directs the recipient's attention towards the task at hand; conversely, a feedback tool that shifts the recipient's attention away from the task will be less effective. The variation of the effectiveness of A/F reports reported in the literature [2] may be partially explained by variation in this characteristic. Characteristics of feedback that direct the attention towards the task which have been shown to be associated with more effective feedback include individualized, frequent, and nonpunitive feedback [5, 19]. In addition, provision of correct solution information (feedback that helps recipients understand what needs to change in order to improve performance) [20] and goal setting [2, 21] are also associated with effectiveness.

Participants had a clear idea of who would likely be underperformers: older, surgically trained physicians who perform a low volume of colonoscopies relative to their younger and/or more specialized peers. They also implied that the solutions for low-performing physicians could differ depending on their career stage. For example, further training could be provided to young, underperforming physicians, while older physicians could be offered incentives to retire.

However, it should be noted that our findings pertain to endoscopists'* perceptions* of poor performers but do not necessarily indicate that older, surgically trained physicians are indeed underperforming relative to their younger peers. It has been reported that one professional group will tend to see another in narrow terms and often attribute tensions and even conflicts about goals and values to these perceived differences [22]. Many of the participants in our study made statements that suggested they imagined that poor performers as being “other” in some ways to themselves.

Our study had some limitations. The participants were experts in their field and were identified through the professional networks of the investigators. Most were well established in their careers, considered opinion leaders, with years of professional experience. Physicians from rural and remote jurisdictions were underrepresented among the interviewed cohort. Therefore, the views of our participants may not be broadly representative of all physicians who perform colonoscopies in Ontario. Additionally, the reactions that we elicited were based on a report populated with simulated data; therefore, the responses/quotes elicited are hypothetical and are removed from the participants' personal experience and professional practice. Reports populated with real data may elicit different reactions. In the future, we plan on eliciting reactions to the endoscopist A/F report from “everyday endoscopists” in Ontario using real data.

## 5. Conclusions

Findings from our study highlight the tension between the exceptional autonomy that physicians have historically had in their practice and more recent concerns with public accountability in the health care system [23]. This is an ongoing tension that has important implications for both policy and practice. Future research should explore from both provider and patient perspectives how discourses and practices of accountability are taken up across various settings. In our study there was agreement that the use of A/F has the potential to improve colonoscopy performance. However, our findings indicate that this intervention must be implemented thoughtfully such as by addressing cognitive dissonance, directing attention towards the task at hand, and ensuring access to resources to help improve colonoscopy performance.

## Supplementary Material

These supplementary quotes, while by no means exhaustive, provide additional examples of how the themes developed in this manuscript were grounded in the interview data.

## Figures and Tables

**Table 1:** Participant characteristics.

Interviewee	Sex	Years in practice	Specialty	Type of practice
1^*∗*^	M	20	Gastroenterologist	Academic
2^*∗*^	M	18	Gastroenterologist	Academic
3	F	<4	General surgeon	Community
4^*∗*^	M	30	General surgeon	Community
5^*∗*^	M	18	Gastroenterologist	Community
6	F	13	Gastroenterologist	Community
7^*∗*^	M	18	General surgeon	Community
8^*∗*^	M	23	Gastroenterologist	Community
9	F	30	Gastroenterologist	Academic
10	M	27	General surgeon	Academic
11^*∗*^	M	20	Gastroenterologist	Academic

^*∗*^Member of the Ontario Colonoscopy Expert Panel, Cancer Care Ontario.
