# Supplementary material for: How to Make Feedback More Effective? Qualitative Findings from Pilot Testing of an Audit and Feedback Report for Endoscopists
Source: Can J Gastroenterol Hepatol. 2016 Sep 18;2016:4983790. doi: 10.1155/2016/4983790 (PMC5045985; doi:10.1155/2016/4983790)
Supplement: Supplementary file 1 — These supplementary quotes, while by no means exhaustive, provide additional examples of how the themes developed in this manuscript were grounded in the interview data. [file 4983790.f1.pdf]

Table of supplementary quotes for the 4 themes

Theme	Additional quotations from interviews
**Overall perceptions of the audit and feedback report**	• *Well, you always wonder how you're performing. You know what the standards are supposed to be but there's no easy way for any of us to keep our own data, and you want to know that you're doing a good job, or if you're doing a bad job where you're doing a bad job... this kind of feedback is so hard to come by in practice. And it's just nice to know where you stand in relation to your peers and in relation to where you're supposed to be.* (General surgeon, Interviewee #3)<br><br>• *…doctors are pretty intense people so… if they're in the lowest, they're[not] going to be very happy, so you might get a complaint from a few people in the lowest, but I think most people are accepting of these quality indicators…What they might argue with is either the definition or the accuracy of it...* (Gastroenterologist, Interviewee #2)
**Accountability and consequences for poor performance**	• *I am not concerned about me receiving it, I'm not concerned about anybody else receiving it. As far as I'm concerned this kind of stuff can be public. (General surgeon, Interviewee #10)*
**Motivation to change or improve skills**	• *... it's difficult because it means that you have to self-reflect and make changes, and many people... many physicians are, unfortunately, too arrogant to accept that they could improve their practice, or that they'd actually have to work to get re-training…you have to be able to self-reflect, and physicians don't always do that very well.* (Gastroenterologist, Interviewee #6)<br><br>• *…the itinerant half-way retired practitioner who's doing this kind of procedure to avoid drawing on their RSP, you know? A 72-year-old person with thirty years of experience who's working one or two days a month, barely making that number… They're not going to be even the least bit interested in changing, and you're just going to have to... and that's the reality, that you're going to just have to let them be leeched out of the system like a spleen…* (General surgeon, Interviewee #7)
**Training for performance enhancement and available resources**	• *…so then what do they do with the information? You might want to have a link of some sort to, "You're not doing so well in this area, here are some options for additional training or extra... you know, reading isn't going to help you do better colonoscopy, so you might consider doing a train-the-trainer program, you might consider doing a preceptorship with the following people," like, somewhere they get educational options that would allow them to improve in a non-judgmental way.* (Gastroenterologist, Interviewee #9)
